# Nanostructured Vanadium Dioxide Materials for Optical Sensing Applications

**DOI:** 10.3390/s23156715

**Published:** 2023-07-27

**Authors:** Jongwon Yoon, Woong-Ki Hong, Yonghun Kim, Seung-Young Park

**Affiliations:** 1 Department of Energy & Electronic Materials, Surface & Nano Materials Division, Korea Institute of Materials Science, Changwon 51508, Republic of Korea; kyhun09@kims.re.kr; 2 Center for Scientific Instrumentation, Korea Basic Science Institute, Daejeon 34133, Republic of Korea; parksy@kbsi.re.kr

**Keywords:** vanadium dioxide, phase transition, nanostructure, optical sensing

## Abstract

Vanadium dioxide (VO_2_) is one of the strongly correlated materials exhibiting a reversible insulator–metal phase transition accompanied by a structural transition from a low-temperature monoclinic phase to high-temperature rutile phase near room temperature. Due to the dramatic change in electrical resistance and optical transmittance of VO_2_, it has attracted considerable attention towards the electronic and optical device applications, such as switching devices, memory devices, memristors, smart windows, sensors, actuators, etc. The present review provides an overview of several methods for the synthesis of nanostructured VO_2_, such as solution-based chemical approaches (sol-gel process and hydrothermal synthesis) and gas or vapor phase synthesis techniques (pulsed laser deposition, sputtering method, and chemical vapor deposition). This review also presents stoichiometry, strain, and doping engineering as modulation strategies of physical properties for nanostructured VO_2_. In particular, this review describes ultraviolet-visible-near infrared photodetectors, optical switches, and color modulators as optical sensing applications associated with nanostructured VO_2_ materials. Finally, current research trends and perspectives are also discussed.

## 1. Introduction

**Figure 1:**
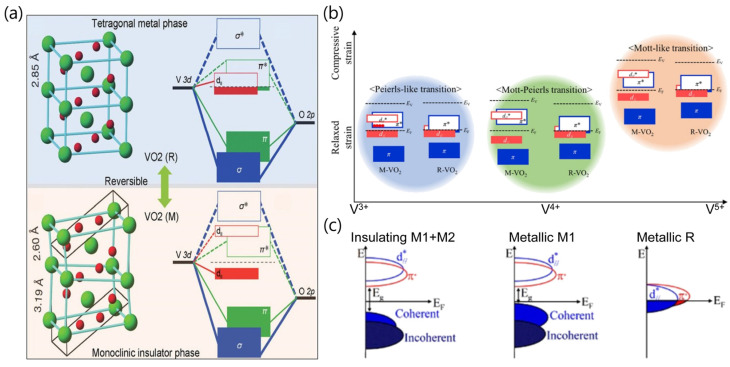
(**a**) Schematic of crystal and electronic band structures of VO_2_ in the high-temperature metallic rutile (R) phase and the low-temperature insulating monoclinic (M) phase. In VO_2_ (R), V^4+^ ions occupied the corner and center positions and each V^4+^ is surrounded by six O^2−^, where the closest V–V distance is approximately 2.85 Å in chains along the c-axis. In VO_2_ (M), the unit cell is a distorted rutile structure of VO_2_ (R) and two different V–V distances of 3.19 and 2.60 Å between the nearest V atoms form the zigzag atom chains. Reproduced with permission from [2], Copyright 2017, WILEY-VCH Verlag GmbH & Co. KGaA, Weinheim. (**b**) Schematic diagram of band structures depicting Peierls, Mott, and collaborative Mott-Peierls transitions. Reproduced with permission from [6], Copyright 2020, American Chemical Society. (**c**) Band structures corresponding to M1, M2, and R phases. Reproduced with permission from [8], Copyright 2020, American Chemical Society.

The complex interplay between charge, spin, orbital, and lattice degrees of freedom results in the novel electronic and magnetic phenomena in strongly correlated materials (SCMs), as an interesting class of materials in condensed-matter physics [1]. Among SCMs, vanadium dioxide (VO_2_) has attracted considerable attention, due to the reversible and dramatic changes in conductance and transmittance during metal–insulator transition (MIT), which is a first-order phase transition accompanied by a crystal structure change from a low-temperature monoclinic phase to a high-temperature rutile phase at near-room-temperature (Tc ~ 340 K), as shown in Figure 1a [2,3]. VO_2_ is a tetragonal rutile (R) structure with space group P4_2_/mnm and lattice constants a = b ≈ 4.55 Å and c ≈ 2.85 Å above Tc, whereas it is a monoclinic M1 structure with space group P2_1_/c and lattice constants a ≈ 5.75 Å, b ≈ 4.53 Å, c ≈ 5.38 Å, b = 122.6° [4]. According to the band theory proposed by Goodenough, the vanadium (V) 3d orbitals are split into σ* (e_g_) symmetry and π* (t_2g_) symmetry states, and the t_2g_ states are further split into two d_π_ orbitals and one d_‖_ orbital [5]. In the R structure, the Fermi level falls between the π* band and the d_‖_ band, whereas in the monoclinic structure, the d_‖_ band is split into two energy bands (d_‖_ and d_‖_*), and a forbidden band with the bandwidth of approximately 0.7 eV between the d_‖_ band and the π* band is formed [5].

The driving mechanisms behind the MIT in VO_2_ have been a topic of controversy for decades whether the transition is driven by electron–electron correlations (Mott transition) or by a structure distortion (Peierls transition). Recently, a collaborative Mott-structural transition mechanism in the phase-transition process has also been proposed as an alternative to the two abovementioned mechanisms of the MIT, because both the structural and electron-correlation aspects are important for describing the MIT behavior in VO_2_ [6,7]. Park and co-workers studied a series of epitaxial VO_2_ films with different deposition temperatures to understand the cooperation effect between Peierls and Mott transitions in VO_2_ [6]. They proposed the diagram of band structures, which provides insights into the role of the strain and multivalent V states on the phase transition of VO_2_, as shown in Figure 1b [6]. In addition, they inferred electronic band structures corresponding to insulating M1 + M2 coexisting phases and metallic M1 and R phases, on the basis of experimental results through hydrogen incorporation in VO_2_, as shown in Figure 1c [8].

**Figure 2:**
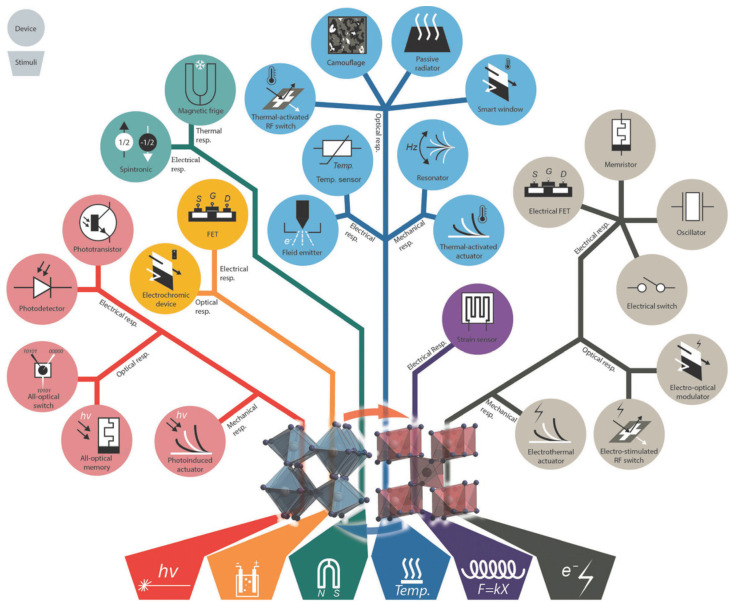
VO_2_-based device applications related to the category of input stimuli and output response. Reproduced with permission from [9], Copyright 2018, WILEY-VCH Verlag GmbH & Co. KGaA, Weinheim.

Although the MIT mechanism is still unclear, the modification of the phase transition and the manipulation of physical properties in VO_2_ are possible by a variety of external stimuli, such as light, temperature, stress, stoichiometry, doping, pressure, electric field, and magnetic field [9]. The distinctive properties of MIT triggered by these stimuli have enabled the demonstration of a wide range of applications shown in Figure 2 [9], such as sensors, switches, smart windows, actuators, memory devices, camouflage, and memristors, including electromagnetic absorption materials [10,11].

In the present review, we focus on emerging optical sensing applications based on nanostructured VO_2_ materials. Firstly, we introduced briefly several synthesis methods of VO_2_ nanostructures and modification techniques of its physical properties. In addition, we describe the potential applications of VO_2_ nanostructures for optical sensing—e.g., photodetectors, optical switches, and color modulators. Finally, the current research trends and prospective research areas of VO_2_ in future applications are also briefly mentioned.

## 2. Synthesis of Nanostructured VO_2_ Materials and Modulation of Their Properties

### 2.1. Synthesis Methods of Nanostructured VO_2_

The morphology of VO_2_ depends on synthesis methods, which are primarily categorized solution- and gas-phase-based synthesis methods. For example, sol-gel process and hydrothermal synthesis are representative solution-based chemical approaches, while pulsed laser deposition (PLD), sputtering method, and chemical vapor deposition (CVD) are gas- or vapor-phase synthesis techniques. In previous reports [3,12,13,14,15], various techniques for the fabrication of nanostructured VO_2_ materials have been described in detail. The advantages and limitations for some of these synthesis methods are summarized in Table 1. Sol-gel or hydrothermal approaches have been used to synthesize nanostructured VO_2_, mainly for the application of thermochromic smart windows. Meanwhile, PLD, sputtering, and CVD have been used to fabricate high quality VO_2_ thin films or single-crystals for the application of MIT-related devices. The various nanostructures (e.g., nanowire, nanorod, nanobeam, nanosheet, nanoparticle, and nanoplate), as well as thin films, can be fabricated by using these synthesis methods. The optical sensing applications based on VO_2_ with different morphologies will be described in Section 3 and, in particular, nanostructured VO_2_-based photodetectors will be summarized in Table 2.

### 2.2. Modulation of Physical Properties of Nanostructured VO_2_

**Figure 3:**
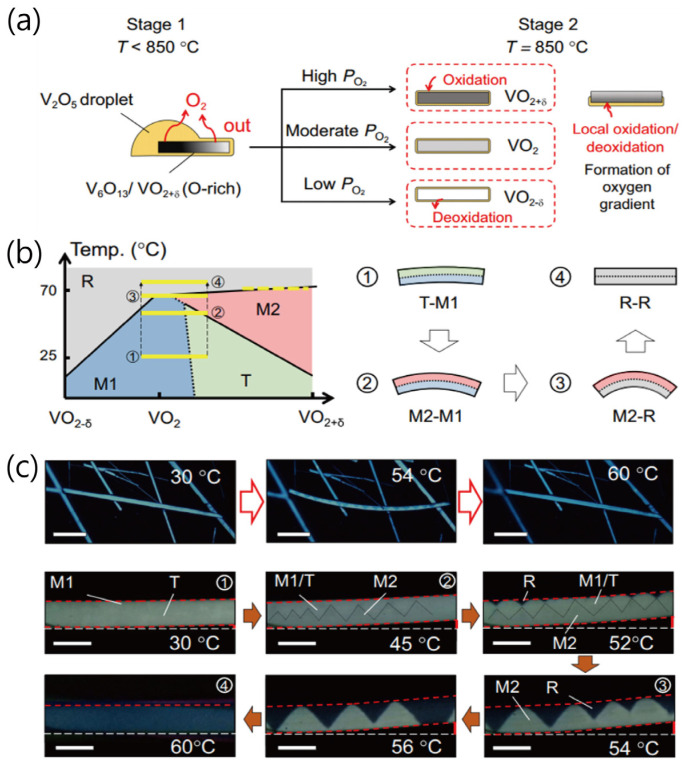
(**a**) Schematics of synthesis process of VO_2_ microbeams in stage 1 (T < 850 °C) and stage 2 (T = 850 °C) under different oxygen partial pressures ($P_{O_{2}}$). (**b**) Working mechanism for a single-crystalline W-doped VO_2_ actuator. The array of yellow gradient bars indicates the traces of actuation process upon heating. (**c**) Optical images of a single-crystalline W-doped VO_2_ actuator at 30, 54, and 60 °C (scale bars are 40 μm). Temperature-dependent optical images of another single-crystalline VO_2_ actuator showing a clear domain evolution process (scale bars are 5 μm). Black dashed lines indicate the positions of domain walls between M2 and M1/T domains. Reproduced with permission from [16], Copyright 2021, Springer Nature.

In recent years, considerable efforts have been devoted to manipulate physical properties (e.g., electrical and optical properties) of nanostructured VO_2_ materials for a variety of applications, such as optical switches, smart window coating, Mott transistors, memristors, sensors, and thermal actuators [12,15]. Most recently, Shi et al. [16] demonstrated the effective phase management of the metallic R phase and insulating phases of monoclinic (M1, M2) and triclinic (T) structures in single-crystalline VO_2_ microbeams through stoichiometry engineering, as shown in Figure 3 [16]. Figure 3a shows the synthesis process of VO_2_ microbeams in the nucleation/growth stage, driven by the reduction of high-valence vanadium precursors (V_2_O_5_ or V_6_O_13_) at T < 850 °C and the stoichiometry-modulation stage for the oxidation or deoxidation of VO_2_ under different partial pressures of oxygen ($P_{O_{2}}$) at T = 850 °C. Using these stoichiometry modulations by adding an appropriate amount of WO_2_, the single-crystalline W-doped VO_2_ actuator with a stoichiometry gradient and selective phase stability was proposed, as shown in Figure 3b. In Figure 3c, the VO_2_ microbeam actuators showed a clear laterally asymmetric configuration and evolution of domains and deflection with increasing temperature. The formation of a radially asymmetric M2-T-M1 domain pattern led to the initial bending at the beginning of the heating stage and with a further increase in temperature, the oxygen-deficient side was gradually occupied by R domains (the oxygen-rich side is occupied by M2 domains). At 60 °C, the entire VO_2_ beam was transformed into the pure R phase of the straight state. As mentioned in ref. [16], the stoichiometry engineering, which was used to selectively stabilize all the three insulating phases (M1, T, M2) in single-crystalline VO_2_ microbeams, may open opportunities for designing and controlling phase inhomogeneity and domains of VO_2_.

**Figure 4:**
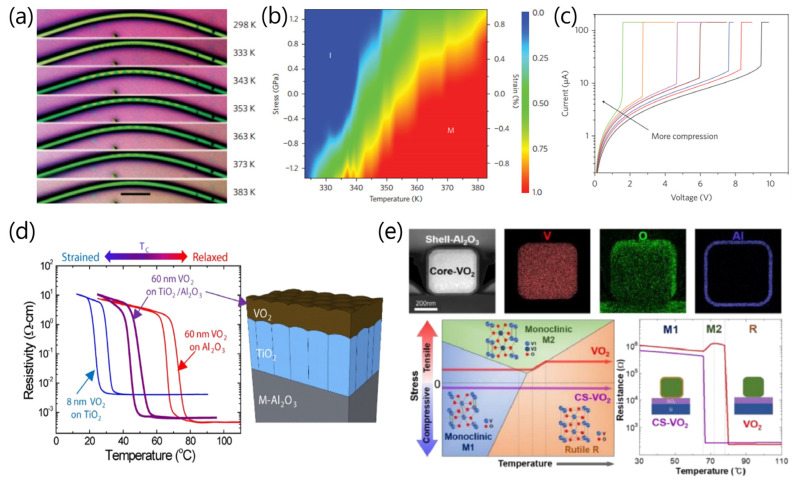
(**a**) Optical images of coexisting triangular metallic (M, dark regions) and insulating (I, bright regions) domains nucleated by strain during heating. (**b**) Phase diagram showing the fraction of the M phase as a function of temperature (*x*-axis) and uniaxial stress (*y*-axis, left) or strain (*y*-axis, right). (**c**) Room-temperature current-voltage characteristic of a VO_2_ microbeam under different axial compressions, showing an MIT induced by Joule heating at different threshold voltages and currents. Reproduced with permission from [19], Copyright 2009, Springer Nature. (**d**) Resistivity-temperature curves for VO_2_ films grown on TiO_2_ and Al_2_O_3_ substrates. Illustration of the VO_2_/TiO_2_/M-Al_2_O_3_ heterostructure. Reproduced with permission from [21], Copyright 2017, American Chemical Society. (**e**) Cross-sectional transmission electron microscopy image and corresponding elemental maps of the rectangular core-shell VO_2_ (CS-VO_2_) nanobeam with a 20-nm-thick shell of Al_2_O_3_. Stress-temperature phase diagram for CS-VO_2_ nanobeams (purple-colored arrow) and pristine VO_2_ nanobeams (red-colored arrow). The arrows show the phase-transition traces on the phase diagram during heating. Temperature-dependent resistance during the heating process for CS-VO_2_ and pristine VO_2_ nanobeams. Reproduced with permission from [22], Copyright 2021, Elsevier.

In addition to stoichiometry engineering, the ability to control domain structures and phase transitions of VO_2_ by strain or stress may lead to a deeper understanding of the correlated electron materials exhibiting the MIT, superconductivity, and magnetoresistance [15,17,18]. Cao et al. [19] demonstrated the manipulation of ordered arrays of metal (M) and insulator (I) domains along single-crystal VO_2_ microbeams by strain engineering, where uniaxial external stress was used to engineer M-I domains and to observe the Mott transition at room temperature, as shown in Figure 4a–c [19]. Figure 4a shows an array of triangle M-I domains which are nucleated and co-stabilized by tensile and compressive strain during heating in a mechanically bent VO_2_ microbeam. In the stress–temperature phase diagram (Figure 4b), when the M phase fraction η = 1 at high temperatures and high compressive stresses, the system was in pure M phase, while it was in pure I phase when η = 0 at low temperatures and high tensile stresses. The coexistence of M and I phases with the spatial arrangement and relative fraction was shown at intermediate temperatures and stresses. In Figure 4c, room-temperature I–V characteristics of a VO_2_ microbeam under different axial compressions show the significant reduction of threshold voltages and currents by the external compression upon MIT, implying the possibility of novel device applications using drastic reduction of the operation power through strain engineering of VO_2_.

The epitaxial VO_2_ nanostructures grown on single-crystal substrates can be strongly affected by the lattice mismatch with substrate or crystal orientations, resulting in determining the relationship between the stress and strain [17,20]. Figure 4d shows resistivity–temperature curves and the surface morphology of VO_2_ films grown on TiO_2_ and Al_2_O_3_ single crystals with various crystallographic orientations [21]. The results show that substrate-dependent strains in the VO_2_ films result in different MIT temperatures. This suggests an enhanced ability to manipulate the MIT properties of VO_2_ by using lattice strain control through the implementation of a microstructured buffer layer in heteroepitaxial oxide thin films. More recently, Shin et al. [22] demonstrated core-shell heterostructure-enabled stress engineering on MIT, providing accommodation of uniform axial stress and control of the phase-transition pathway and properties in VO_2_ nanobeams. In this previous study [22], core-shell VO_2_-Al_2_O_3_ (CS-VO_2_) nanobeams exhibited a simple and direct M1–R phase-transition pathway at a lower temperature without the appearance of metastable intermediate phases (M2 or T), compared to pristine VO_2_ nanobeams with an M1–M2–R transition pathway, as shown in Figure 4e. These results provide the unique insight that the formation of uniform stress states through core-shell architectures can be applied to the design of phase-transition paths and physical properties for VO_2_-based device applications using the MIT process.

**Figure 5:**
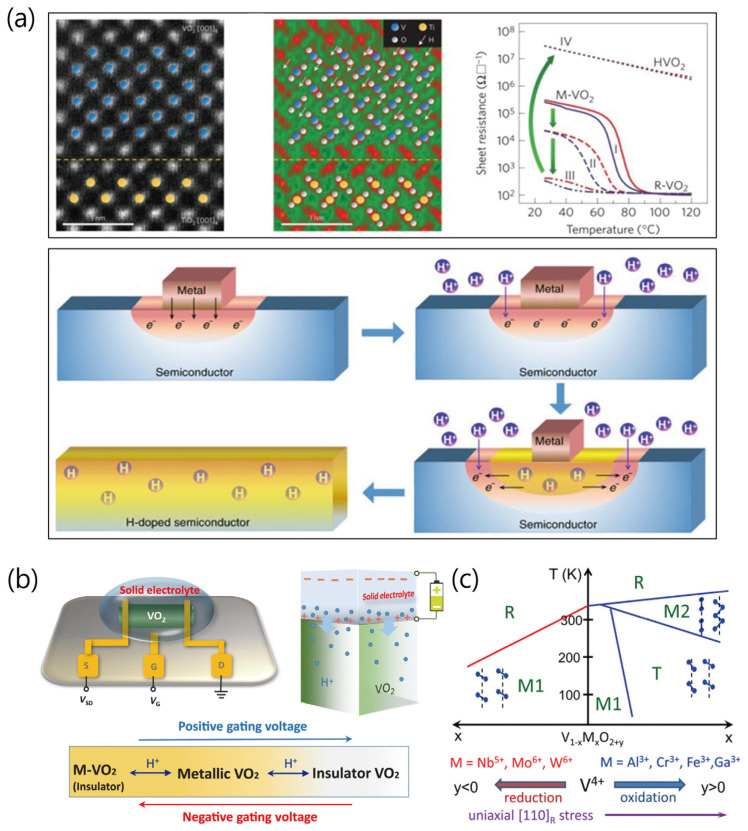
(**a**) Scanning transmission electron microscopy images of the (100) fully hydrogenated HVO_2_ film grown on TiO_2_ (100) substrates and the temperature-dependent sheet resistance in H_x_VO_2_ films with increasing hydrogen content (x) from plot I to plot IV, indicating a transition from insulator (M-VO_2_)-to-metal (H_x_VO_2_)-to-insulator (HVO_2_) (upper panel). A schematic illustration of the contiguous electron–proton co-doping mechanism with the metal-acid treatment of a semiconductor (lower panel). Reproduced with permission from [5], Copyright 2018, Springer Nature. (**b**) The gating diagram and hydrogen ion movement under gating control for the VO_2_ device with source, drain, and gate electrodes. The reversible insulator-metal-insulator tristate phase transitions of VO_2_ by tuning hydrogenating level with positive or negative gating voltages. Reproduced with permission from [24], Copyright 2019, American Association for the Advancement of Science. (**c**) Temperature-composition phase diagram showing influence of doping and uniaxial stress along the [110]_R_ crystallographic direction of the V_1−x_M_x_O_2+y_. Reproduced with permission from [25], Copyright 2012, American Chemical Society.

Meanwhile, doping in VO_2_ has attracted much attention as an effective way for its electrical and optical modulation for electronic and optical device applications [5,23,24,25]. Shao et al. [5] reviewed previous works by Yoon et al. [26] and Zou et al. [27]: (1) a two-step insulator (M-VO_2_)-to-metal (HxVO_2_)-to-insulator (HVO_2_) modulation as the hydrogen concentration increases in nano-sized Pt-island-decorated VO_2_ layers during annealing the samples at 120 °C, under forming gas containing 5% hydrogen gas (Figure 5a, upper panel); (2) a facile approach to hydrogenate monoclinic VO_2_ in an acidic solution under ambient conditions, by placing a small piece of low-work function metal (Al, Cu, Ag, Zn, or Fe) on the VO_2_ surface (Figure 5a, lower panel). Recently, Chet et al. [24] modulated the insertion/extraction of hydrogen into/from the VO_2_ lattice at room temperature through a solid electrolyte-assisted gating control, resulting in tristate phase transitions that enable the control of light transmittance, as shown in Figure 5b. Strelcov et al. [25] proposed a new high-yield method of doping VO_2_ nanostructures with aluminum, which could provide possible stabilization of the monoclinic M2 phase for realization of a purely electronic Mott transition field-effect transistor (Figure 5c). According to previous reports [28,29], uniaxial stress and doping can stabilize the M2 phase at ambient conditions. In the schematic diagram depicting phase transformations of VO_2_ phases by metal-ion dopants (Figure 5c), dopants of higher oxidation states (M = W^6+^, Nb^5+^, and Mo^6+^) lower the transition temperature, whereas dopants of lower oxidation states (M = Cr^3+^, Al^3+^, Fe^3+^, or Ga^3+^) stabilize the M2 and T phases of VO_2_ at room temperature [25,30,31]. This behavior shows the influences of reduction and oxidation of the V^4+^ ions, respectively, in which the oxidation effect is similar to the effect of application of uniaxial stress along the [110] direction of the R phase [25].

## 3. Optical Sensing Applications

**Figure 6:**
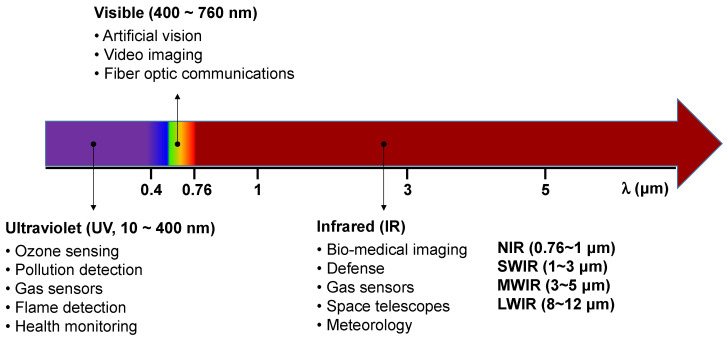
Spectral range for the typical photodetectors, and their potential applications corresponding to each wavelength range (UV, Visible, and IR). IR is divided into four different ranges; NIR (0.76–1 μm), SWIR (1–3 μm), MWIR (3–5 μm), and LWIR (8–12 μm).

In general, the detectable wavelength ranges for photodetectors are categorized as ultraviolet (UV, 10–400 nm), visible (400–760 nm), near-infrared (NIR, 760–1000 nm), short-wavelength infrared (SWIR, 1–3 μm), mid-wavelength infrared (MWIR, 3–5 μm), and long-wavelength infrared (LWIR, 8–12 μm) [32]. According to these detectable ranges, photodetectors can be used in a variety of potential applications, as shown in Figure 6. VO_2_ has an optical bandgap (~0.7 eV) and high-temperature coefficient of resistance (TCR), suggesting its potential for optical sensing applications over a wide wavelength range. In addition, the MIT of VO_2_ allows optical switching in the IR wavelength range and color modulation. In this section, we describe the representative photodetection mechanism and review up-to-date studies for VO_2_-based photodetectors. Other optical applications of transmission and color modulation are briefly reviewed.

### 3.1. Detection Mechanisms for VO_2_-Based Photodetectors

#### 3.1.1. Light-Induced Phase Transition

VO_2_ typically has a phase-transition characteristic from the insulating M1 phase to metallic R phase above critical temperature (T_C_) of approximately 67 °C. Instead of thermal sources, optical excitation allows the phase transition of VO_2_ to occur on a picosecond time scale. However, the metallic state is not permanent and is transformed back into the insulating state [33,34]. Unlike a temporary phase transition, exposure of UV light on a VO_2_ can induce a permanent phase transition from an insulating to a metallic state [35]. Li et al. [36,37] also reported that a longer UV exposure duration led to a greater reduction in the resistance of the VO_2_ film, suggesting the possibility of UV detection. It is believed that photo-induced oxygen vacancies can induce an electronic phase transition leading to electrical resistance changes in the VO_2_ film during UV exposure.

#### 3.1.2. Photoconductive Effect

By the incident photons with the energies greater than the energy bandgap (E_g_), the electrical conductivity of the conduction channel can be modulated owing to the generation of electron-hole pairs, which change the carrier concentration in the conduction channel. The electron-hole pairs are separated by the applied electric field, generating a photocurrent (I_ph_). I_ph_ depends on the applied potential, charge carrier mobility, and carrier lifetime. Because the reported E_g_ value of monoclinic VO_2_ was approximately 0.7 eV, the photoconductive effect can be a dominant detection mechanism for the photodetectors using a VO_2_ as a conductive channel under UV to NIR illumination

#### 3.1.3. Photogating Effect

Because VO_2_ nanostructures have a large surface-to-volume ratio, oxygen molecules adsorbed on the surface can significantly affect their electrical conductivity by acting as electron acceptors. Under UV illumination, the adsorbed oxygen molecules are desorbed from the surface by recombination with photo-generated holes. Such absorption and desorption of oxygen molecules on the surface of VO_2_ nanostructures under illumination can modulate the carrier concentration. The incident light acts as a gate for carrier modulation (photogating effect).

#### 3.1.4. Photovoltaic Effect

When the incident photon energy is higher than the E_g_ of the semiconductor materials, electrons are excited from the valence band to the conduction band, generating electron-hole pairs. Such photoexcited carriers are driven by a built-in electric field arising from semiconductor–semiconductor or semiconductor–metal junctions such as p-n junctions and Schottky junctions. Because a VO_2_ (M1) with an insulating phase is considered an n-type semiconductor, the formation of contacts with other semiconductor materials enables photodetection via the photovoltaic effect. Recently, heterojunctions between 2D materials and VO_2_ have been employed for photodetectors using the photovoltaic effect; the examples are explained in the following section.

#### 3.1.5. Photobolometric Effect

The absorption of photon energy can induce resistance changes in materials by increasing temperature. The figure-of-merit of the photodetector based on the photobolometric effect is the TCR, where TCR = dR/R·dT. It is well known that VO_2_ has high TCR (−4%·K^−1^) and such a property enables commercial IR photodetectors (microbolometer) [38]. In VO_2_-based photodetectors, the photobolometric effect is believed to be dominant in the MWIR and LWIR regions.

### 3.2. VO_2_-Based Photodetectors

#### 3.2.1. Ultraviolet (UV) Photodetection

**Figure 7:**
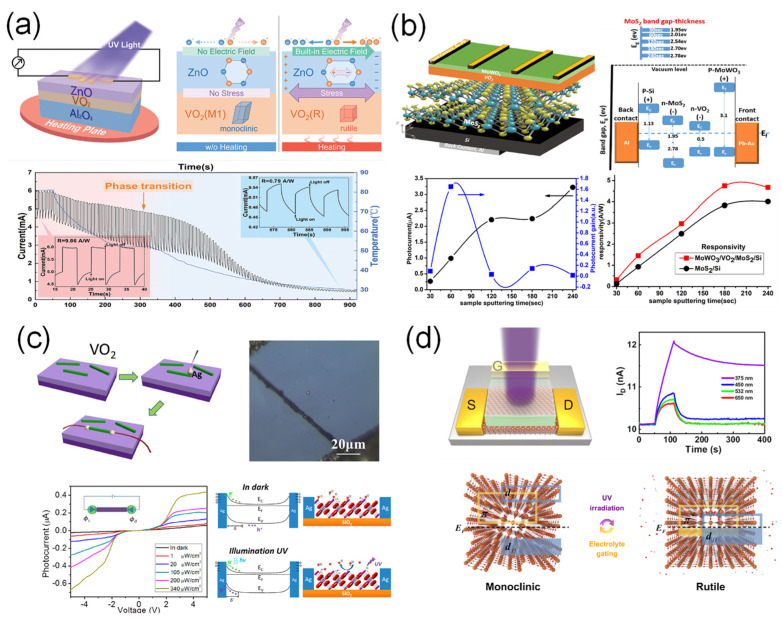
VO_2_-based UV photodetectors. (**a**) ZnO/VO_2_-heterojunction-based UV photodetectors using phase-transition-induced tensile stress in ZnO thin film. Reproduced with permission from [39], Copyright 2020, Royal Society of Chemistry. (**b**) VO_2_/MoS_2_/Si multilayer-based UV photodetectors using energy-band engineering. Reproduced with permission from [40], Copyright 2020, Springer Nature. (**c**) VO_2_ microwire-based UV photodetector using a photogating effect. Reproduced with permission from [41], Copyright 2014, American Chemical Society. (**d**) VO_2_ thin film-based UV photodetector via UV-induced phase transition. Reproduced with permission from [37], Copyright 2022, Springer Nature.

The structural change in VO_2_ from the M1 to the R phase at the phase-transition temperature can induce lattice expansion, leading to tensile stress in the interfaced film. Xin et al. [39] reported a UV photodetector based on a ZnO/VO_2_ heterostructure with a high responsivity in the UV range (Figure 7a). In this device architecture, the photodetectors exhibited high photoresponsivity and a fast photoresponse at the phase-transition temperature of the VO_2_ thin film. The structural phase transition of VO_2_ can induce tensile stress in ZnO thin films owing to lattice expansion. In addition, it is well known that ZnO has piezoelectric properties and that polarization can occur in the crystal. Therefore, the phase transition of VO_2_ could lead to an internal electric field in ZnO thin films. Such an electric field boosted the charge separation of the electron-hole pairs generated by UV illumination, resulting in a high photoresponsivity (R = 10.07 A·W^−1^) and a faster photoresponse (rise = 0.020 s, decay = 0.032 s), compared to the device below the phase-transition temperature (R = 0.32 A·W^−1^, rise = 2.49 s, decay = 2.06 s).

Basyooni et al. [40] reported a UV photodetector with a vertically stacked device configuration consisting of a VO_2_/MoS_2_/Si thin film and asymmetric metal contacts for energy-band alignment (Figure 7b). Here, the insertion of the VO_2_ thin film enabled higher conductivity and photocurrent owing to the high carrier mobility and enhanced photon absorption characteristics, compared to the MoS_2_/Si device. Consequently, an increase in the photoresponsivity and specific detectivity was observed in the VO_2_/MoS_2_/Si heterostructure. The maximum photoresponsivity of the device was 4.7 A·W^−1^.

Employing nanostructures into the photodetectors is promising due to their large surface-to-volume ratio, which allows for enhanced light absorption and response. Wu et al. [41] demonstrated a UV photodetector using a single VO_2_ microwire with a one-dimensional (1D) structure (Figure 7c). It is well known that a 1D structure has a relatively large surface-to-volume ratio, which significantly affects the sensing characteristics of devices. In particular, oxygen molecules can be easily adsorbed on the surface of the VO_2_ microwire, capturing conduction electrons. This can induce surface depletion of the VO_2_ microwire. When illuminated with UV light, electron-hole pairs can be generated, and the holes can migrate to the surface and recombine with the adsorbed oxygen molecules, reducing surface depletion. As a result, the device showed a significant enhancement in detection performance owing to the photogating effect. The reported photoresponsivity of the device was approximately 7069 A·W^−1^.

UV exposure of VO_2_ can induce a phase transition as described previously. Li et al. [36,37] reported that UV illumination at an intensity of 64 mW·cm^2^ caused the nonvolatile and gradual conductance change in a VO_2_ by the phase transition, while the illumination of visible light induced volatile conductance change in a VO_2_ due to rapid recombination after turning off the light, as shown in Figure 6d. Based on these unique optoelectronic properties, the authors demonstrated the application of artificial synaptic devices.

#### 3.2.2. Visible Photodetection

**Figure 8:**
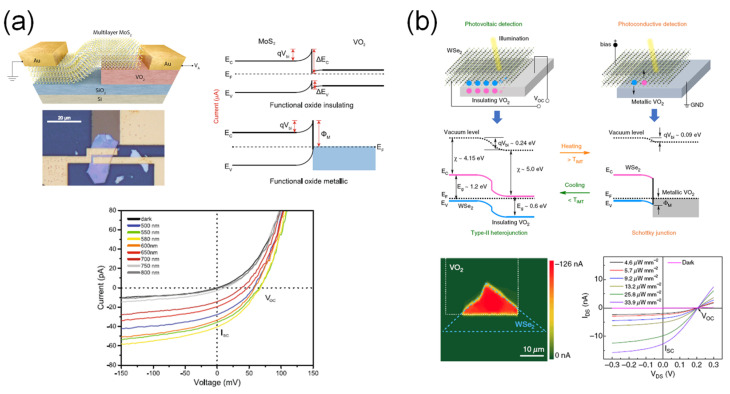
VO_2_-based visible photodetectors. (**a**) MoS_2_/VO_2_-heterojunction-based visible photodetector by using photovoltaic effect. Reproduced with permission from [42], Copyright 2017, Springer Nature. (**b**) WSe_2_/VO_2_ heterojunction-based visible photodetector by using both photovoltaic and photoconductive effects. Reproduced with permission from [43], Copyright 2019, American Institute of Physics.

Regarding VO_2_-based photodetectors, interfacing with two-dimensional (2D) materials enables photodetection in the visible range because of the E_g_ of the 2D materials. Oliva et al. [42] demonstrated a photodetector based on a MoS_2_/VO_2_ heterojunction (Figure 8a). The heterojunction exhibited rectification behavior due to the energy band alignment, leading to a lower leakage current in the reverse bias region. In addition, it showed a relatively higher photoresponsivity in the visible range than multilayer MoS_2_ devices reported in other studies. The maximum photoresponsivity of the device was approximately 1.25 A·W^−1^. Luo et al. [43] introduced a WSe_2_/VO_2_ heterojunction to form a p-n junction, as shown in Figure 8b. The fabricated photodetector showed dual-mode operation depending on the VO_2_ phase transition temperature. At room temperature, VO_2_ had an insulating phase, and the photodetector exhibited photovoltaic properties due to the built-in potential formed by the p-n junction. However, when VO_2_ had a metallic phase at 90 °C, the photodetector was operated via photoconductive effect, forming a Schottky contact between WSe_2_ and VO_2_. The WSe_2_/VO_2_ photodetector indicated a relatively high photoresponsivity of 2.4 A·W^−1^ at room temperature and 6.6 A·W^−1^ at 90 °C.

#### 3.2.3. Near-IR (NIR) Photodetection

**Figure 9:**
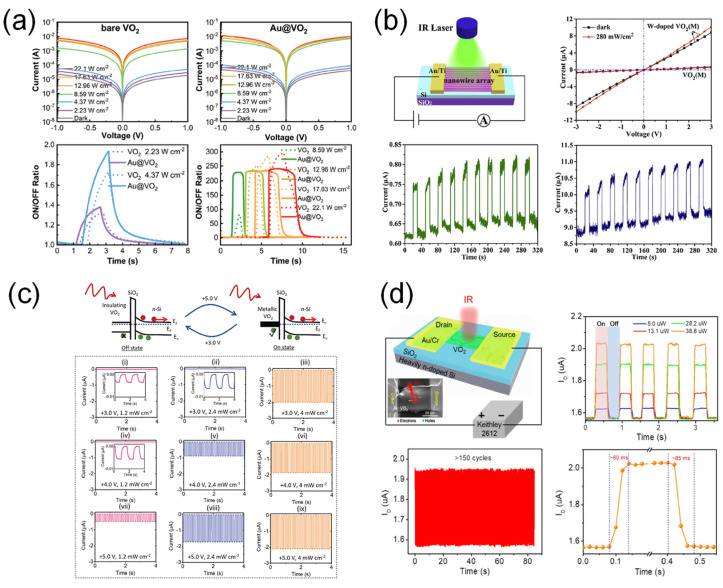
VO_2_-based NIR photodetectors. (**a**) Au nanoparticle-decorated VO_2_-based NIR photodetectors using an LSPR effect. Reproduced with permission from [44], Copyright 2021, WILEY-VCH Verlag GmbH & Co. KGaA, Weinheim. (**b**) NIR photodetectors based on W-doped VO_2_ nanowire arrays using photoconductive effect. Reproduced with permission from [45], Copyright 2018, Elsevier. (**c**) VO_2_/p-Si-heterojunction-based NIR photodetector. Phase transition of VO_2_ by applied external bias and NIR illumination allows the efficient hole collection as shown in the energy band diagram. Reproduced with permission from [48], Copyright 2023, WILEY-VCH Verlag GmbH & Co. KGaA, Weinheim. (**d**) NIR photodetector based on a CVD-grown VO_2_ thin film. The device was operated by photoconductive effect under NIR illumination (λ = 850 nm). Reproduced with permission from [49], Copyright 2021, Springer Nature.

Recently, a VO_2_-based NIR photodetector using the localized surface plasmon resonance (LSPR) effect was demonstrated to enhance the photodetector performance, as shown in Figure 9a [44]. The device showed significant enhancement in I_ph_ under the illumination of light (λ = 808 nm, P = 8.59 W·cm^2^) due to the MIT property and LSPR effect leading to the hot electron injection. The fabricated NIR photodetector showed the maximum photoresponsivity of 502.1 mA·W^−1^.

Xie et al. [45] reported the highly ordered W-doped VO_2_ nanowire arrays for NIR detection (Figure 9b). One-dimensional nanowire arrays increased the effective area for photon absorption. In addition, it was reported that the E_g_ of W-doped VO_2_ was smaller than that of bare VO_2_ [46], and doped W could prevent the recombination of electron-hole pairs, thereby extending the exciton lifetime [47]. As a result, the W-doped VO_2_ nanowire arrays showed a much higher photocurrent and photoresponsivity than the bare VO_2_ nanowire arrays. The photoresponsivity of the W-doped VO_2_ nanowire array and bare VO_2_ nanowire array were 21.4 mA·W^−1^ and 0.29 mA·W^−1^, respectively.

A VO_2_/n-Si heterojuction for NIR photodetection, which showed low dark current and linear photoresponse characteristics, was also demonstrated as shown in Figure 9c [46]. In particular, enhanced photoresponsivity and a faster photoresponse were observed at relatively high electric fields and optical power densities. The enhanced optoelectronic performance could be resulted from the MIT of VO_2_ by the applied electric field and NIR illumination (λ = 940 nm), leading to the efficient collection of the photoexcited electron-hole pairs in n-Si, as shown in the energy band diagram in Figure 9c. The maximum photoresponsivity was 1.01 mA·W^−1^.

Guo et al. [49] demonstrated an NIR photodetector based on a VO_2_ film synthesized via CVD in which a VO_2_ film was used as the photoconductor (Figure 9d). When the NIR light (λ = 850 nm) was illuminated on the VO_2_ channel, a photocurrent was generated by the photoexcited electron-hole pairs and an applied electric field (photoconductive effect). The photoresponsivity of the device was approximately 16 mA·W^−1^.

#### 3.2.4. IR Photodetection

**Figure 10:**
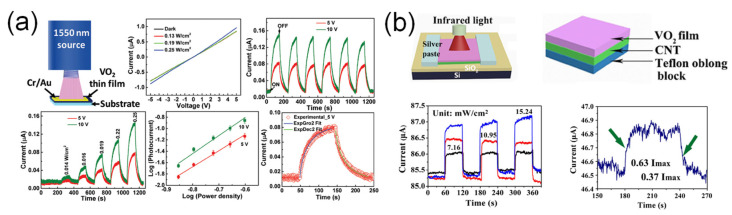
VO_2_-based IR photodetectors. (**a**) VO_2_-based SWIR photodetector using photoconductive effect. Reproduced with permission from [50], Copyright 2020, The Royal Society of Chemistry. (**b**) VO_2_ nanowire/carbon nanotube (CNT) composite-based IR photodetector via photobolometric effect. Reproduced with permission from [51], Copyright 2017, Springer.

Infrared (IR) with wavelength longer than NIR is classified into SWIR (1–3 μm), MWIR (3–5 μm), and LWIR (8–12 μm). Because VO_2_ has a small E_g_ (~0.7 eV) and a high TCR, photoconductive and photobolometric effects are the dominant photodetection mechanisms. Rajeswaran et al. [50] demonstrated a VO_2_-based SWIR photodetector (Figure 10a) and observed its electrical properties under illumination (λ = 1550 nm). Because the absorbed photon energy (~0.8 eV) is higher than the E_g_ of VO_2_, the photoexcited electron-hole pairs can be generated and contribute to the photocurrent of the device. At high optical power density and applied bias region, the device showed a high photoresponse and then the maximum photoresponsivity was 7.13 × 10^−2^ mA·W^−1^.

Fu et al. [51] fabricated the photodetector based on vertically stacked 1D VO_2_ nanowire/carbon nanotube (CNT) composite film (Figure 10b). The composite-film-based photodetector exhibited both enhanced photoresponsivity and a faster photoresponse than the VO_2_-nanowire-based photodetector. The CNT film played a role as a medium for heat absorption and transfer between the CNT and VO_2_ films, leading to improved IR response characteristics. The photoresponsivity of the device was 17.83 mA·W^−1^. Ma et al. [52] used a VO_2_/silicon nitride (SN) composite film for IR photodetection. A flexible and freestanding thin film photodetector was fabricated using SN nanotubes. The device showed strong IR absorption and low heat capacity, leading to enhanced IR photodetection.

#### 3.2.5. Broadband Photodetection

**Figure 11:**
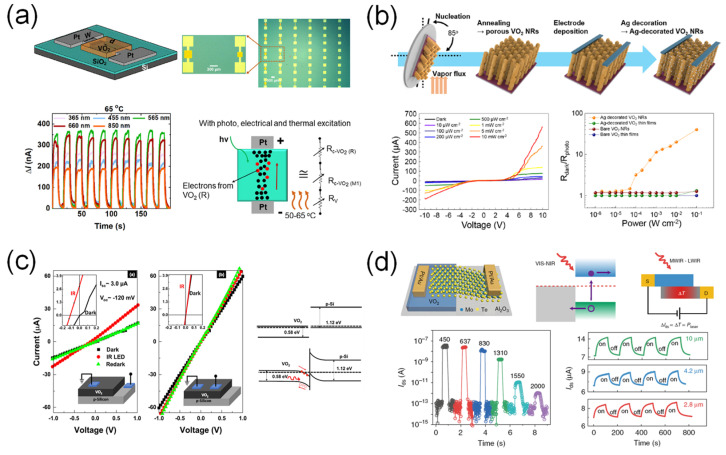
VO_2_-based broadband photodetectors. (**a**) Broadband photodetector that is detectable from UV to NIR. Such a photodetection property contributes to the photo-excitation, electrical excitation, and thermal excitation, simultaneously. Reproduced with permission from [53], Copyright 2020, Elsevier. (**b**) Vertically aligned and Ag-decorated VO_2_ nanorod-based broadband photodetector allowing detection from visible to NIR using LSPR effect by Ag nanoparticles. Reproduced with permission from [55], Copyright 2019, American Chemical Society. (**c**) VO_2_/p-Si heterojunction-based broadband photodetector (visible to NIR) using photovoltaic effect. The p-n junction forms the built-in potential leading to the efficient separation of photoexcited carriers. Reproduced with permission from [56], Copyright 2022, Elsevier. (**d**) VO_2_/MoTe_2_ heterojunction-based broadband photodetectors with dual-mode operation. The device operates by photovoltaic effect in visible-to-SWIR range, and by photobolometric effect in MWIR-to-LWIR range. Reproduced with permission from [57], Copyright 2020, Springer Nature.

Recently, VO_2_-based broadband photodetectors have been intensively studied due to their potential applications. Kabir et al. [53] demonstrated a broadband photodetector based on a VO_2_ thin film synthesized by DC sputtering and annealing in ambient air (Figure 11a). According to the literature, the broadband photodetection of a device is attributed to the photo-excitation, electrical excitation, and thermal excitation, simultaneously. In particular, the enhanced photoresponse after the phase transition from the insulating to the metallic phases of VO_2_ is attributed to free carriers. The highest photoresponsivity of the device was approximately 2 A·W^−1^ at the metallic phase of VO_2_ and in the visible range. Umar et al. reported similar device configurations but used different metal contacts (Ag). The authors suggested that the photocurrent was generated by a photon energy higher than the E_g_ of VO_2_ [54].

Hong et al. [55] introduced a glancing angle deposition method to form vertically aligned VO_2_ nanorods, as shown in Figure 11b. The porous VO_2_ nanorods provided a wide specific area for light absorption. In addition, Ag-nanoparticle-decorated VO_2_ was employed to induce an LSPR effect. The electric field at the interface between the Ag nanoparticles and VO_2_ nanorods enabled the broadband photodetector (visible to NIR) and enhanced the photoresponse of the device. Interestingly, the device showed high photoresponsivity from visible to NIR ranges and the maximum photoresponsivity was approximately 10^3^ A·W^−1^ in the NIR range.

Hassan et al. [56] demonstrated a photodetector employing a vertical VO_2_/p-Si heterojunction (Figure 11c). The formed p-n junction enabled the self-powered operation of the photodetectors. The excited photocarriers could be separated using the built-in electric field of the device. The maximum photoresponsivity of the photodetector was approximately 0.02 mA·W^−1^. Jiang et al. [57] introduced a VO_2_/MoTe_2_ heterojunction into a photodetector to form a p-n junction (Figure 11d). The fabricated photodetector exhibited broadband photodetection properties in the visible-to-SWIR range. In addition, due to the high TCR of the VO_2_ film, the MWIR and LWIR were detectable. The photoresponsivity observed in the NIR range (λ = 830 nm) was approximately 0.22 A·W^−1^.

### 3.3. Summary of VO_2_-Based Photodetectors

Table 2 shows a summary of the up-to-date VO_2_-based photodetectors. In this table, we summarize the materials, detectable wavelengths, deposition methods for VO_2_, photoresponsivity (R), and specific detectivity (D *) of the photodetectors.

**Table 2:** Summary of VO_2_-based photodetectors.

Materials Type	λ (nm)	Methodology	R (A/W)	D * (Jones)	Ref.
VO_2_	375	PLD	-	-	[36,37]
VO_2_/Nb-doped TiO_2_	254/405	DC sputter	15.7 (@ 405 nm) 35.6 (@254 nm)	-	[58]
MoWO_3_/VO_2_/ MoS_2_/Si	365	RF sputter	4.7	4.3 × 10^8^	[40]
VO_2_ (1D)	360–400	CVD	7069	1.5 × 10^14^	[41]
ZnO/VO_2_	365	PLD	10.07	1.2 × 10^10^	[39]
VO_2_/MoS_2_	500–700	DC Sputter	1.25	-	[42]
VO_2_/WSe_2_	532	DC Sputter	2.4 (@ RT) 6.6 (@ 90 °C)	1.9 × 10^13^ (@ RT) 1.8 × 10^11^ (@ 90 °C)	[43]
Au/VO_2_	808	DC Sputter	0.26	1.14 × 10^11^	[44]
W-doped VO_2_ (1D)	980	Hydrothermal	0.02	-	[45]
VO_2_/n-Si	940	ALD	0.001	1.0 × 10^12^	[48]
H-doped VO_2_ nanoparticles	780	Sol-gel	3.6 × 10^4^	1.1 × 10^13^	[59]
VO_2_/p-Si	850	RF sputter	14.8	7.0 × 10^12^	[60]
VO_2_	850	CVD	0.02	-	[49]
VO_2_	1550	CVD	7.1 × 10^−5^	1.1 × 10^11^	[50]
VO_2_	1064/1550	DC sputter	0.014 (@ 1064 nm)	1.7 × 10^12^ (@ 1064 nm)	[61]
VO_2_ (1D)/CNT	IR	Hydrothermal	0.6 × 10^−3^	-	[51]
VO_2_/ZnO	365/525/1064	Hydrothermal	0.5 × 10^−3^ (@ 365 nm)	2.7 × 10^9^ (@ 365 nm)	[62]
VO_2_	365–850	DC sputter	0.9 (@ 565 nm, M1) 2.1 (@ 850 nm, R)	9.4 × 10^9^ (@ 565 nm, M1) 4.6 × 10^9^ (@ 850 nm, M1)	[53]
VO_2_/Si	650/980	PLD	0.35 (@ 650 nm)	-	[54]
VO_2_/MoTe_2_	450–2000, 2800–10,000	DC sputter	0.22 (@ 830 nm)	3.0 × 10^10^ (@ 830 nm)	[57]
VO_2_/p-Si	456,515,950	PLD	2.0 × 10^−5^ (@ 950 nm)	-	[56]
Ag/VO_2_ (1D)	400–1000	E-beam evaporation	4.1 × 10^3^	1.4 × 10^14^	[55]

### 3.4. Optical Switching and Color Modulator Applications

**Figure 12:**
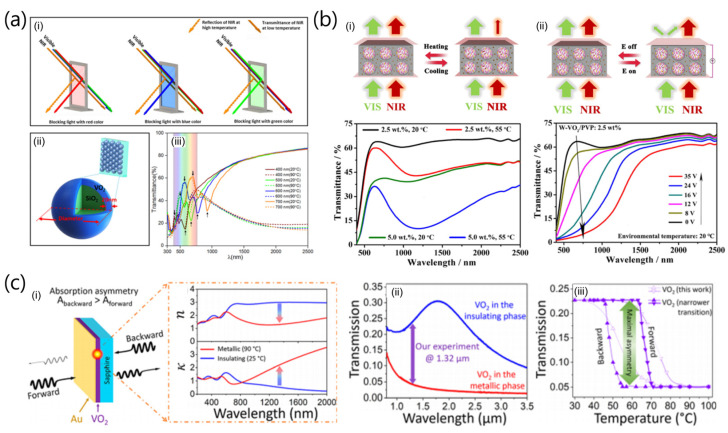
(**a**) (**i**) Working mechanism of photonic VO_2_ smart window; (**ii**) illustration of designed structures for simulation; and (**iii**) simulated transmittance spectrum, where transmittance peaks and troughs are indicated by the solid and dashed arrows, respectively. The colorful background in (**iii**) denotes the visible spectrum from 370 to 770 nm. Reproduced with permission from [63], Copyright 2016, American Chemical Society. (**b**) Schematic illustration of the optical behaviors of the as-prepared film upon the heating or cooling (**i**, upper panel) and by applying or removing the voltage at low environmental temperature (**ii**, upper panel). Vis/NIR transmittance spectra from 400 nm to 2500 nm for the films with W-VO_2_ nanocrystals at 20 and 55 °C (**i**, lower panel), and under the application of various voltages at 20 °C (**ii**, lower panel). Reproduced with permission from [64], Copyright 2017, American Chemical Society. (**c**) (**i**) Schematic of a planar-limiting optical diode made from sputtered VO_2_ and its real and imaginary refractive indices in its insulating (25 °C) and metallic (90 °C) phases. (**ii**) Transmission spectra of the limiting optical diode in the unswitched (blue, insulating) and switched (red, metallic) states at room temperature and 90 °C, respectively. (**iii**) Simulation results, including VO_2_ films with a narrower phase transition [69]. Reproduced with permission from [65], Copyright 2018, American Chemical Society.

VO_2_ has been extensively investigated as a tunable material for optical modulation systems due to the drastic change in the IR transmittance and the refractive index across the MIT [63,64,65]. Long and co-workers demonstrated static visible light tunability and dynamic NIR modulation of two-dimensional SiO_2_-VO_2_ core-shell photonic crystal films, as shown in Figure 12a [63]. The SiO_2_-VO_2_ core-shell photonic crystal-based thermochromic smart window can show the tunable functionality via selectively reflecting and blocking light (indicated by red, blue, orange, and green arrows) and simultaneously maintaining (attenuating) IR transmission at low temperature (at high temperature) (Figure 12(ai)). The finite difference time domain simulations for transmission spectra of the photonic crystal structure predict that the transmittance can be tuned across the visible spectrum, while maintaining good solar regulation efficiency (ΔT_sol_ = 11.0%) and high solar transmittance (T_lum_ = 49.6) (Figure 12(aii,iii)).

Liang et al. [64] presented dual-band modulation of visible and NIR transmittance through voltage and temperature in a hybrid micro–nano composite film, which contains the microsized liquid crystals domains with a negative dielectric constant and tungsten-doped vanadium dioxide (W-VO_2_) nanocrystals (Figure 12b). The light modulation performance of the films with 2.5 and 5.0 wt.% W-VO_2_ nanocrystals showed transparency in the visible region and a drastic change in NIR transmittance at different temperatures (Figure 12(bi)). This result indicates that NIR light transmittance of the hybrid composite film can be passively modulated according to the temperature variations. In addition, the visible light transmittance of the hybrid composite film can be independently and dynamically regulated by the external voltages (Figure 12(bii)). Specifically, Vis/NIR spectra of the films with 2.5 wt.% of W-VO_2_/PVP nanocrystals showed that the visible light transmittance of the film gradually decreased due to a spatial variation of IR between the micro-liquid crystal domains and the polymer during the increase in the applied voltages from 0 to 35 V, resulting from the parallel alignment to the direction of the electric field of liquid crystals (Figure 12(bii)).

Wan et al. [65] demonstrated a VO_2_-based limiting optical diode as a nonlinear device that features asymmetric transmission of light, which was bidirectionally transparent at low power but opaque during illumination of a sufficiently intense light incident from a particular direction. The proof-of-concept of a VO_2_-based limiting optical diode comprising a transparent sapphire substrate, a thin VO_2_ layer, and a semitransparent gold film shows the asymmetric absorption of a VO_2_ thin film to selectively trigger the MIT, enabling asymmetric transmission (Figure 12(ci)). For the case of forward incidence, a significant amount of power is reflected before it reaches the VO_2_, whereas there is substantially more absorption of the light by the VO_2_ in the case of backward incidence. This could be supported by the temperature-dependent infrared refractive indices of the VO_2_ film. Due to a large change in n and κ for a relatively small change in temperature, a VO_2_-based limiting optical diode can be designed to operate over a broad wavelength range (1–3.5 μm) (Figure 12(cii)) and, by using VO_2_ films with narrower transitions, maximal asymmetry can be reached in the simple thin-film geometry (Figure 12(ciii)).

**Figure 13:**
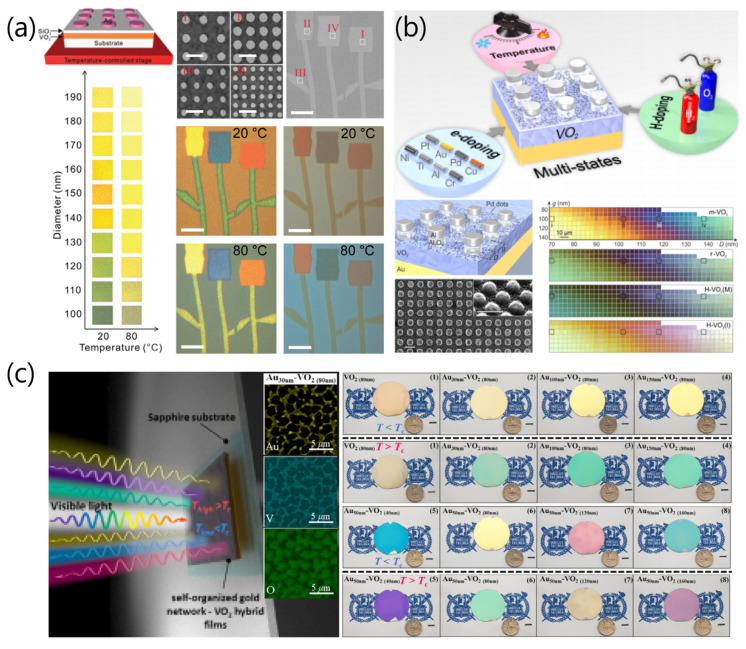
(**a**) Optical properties of VO_2_ film comprising periodic silver-nanodisk array with different diameters. Reflection images of samples with nanodisk diameters ranging from 100 to 190 nm in steps of 10 nm at 20 and 80 °C. Magnified SEM images of the four different pattern-containing regions, where all scale bars are 400 nm in length. SEM image of the patterns (I, II, III, and IV); the sale bar is 40 µm in length. Reflection images of the patterns with different SiO_2_ thickness at 20 °C and at 80 °C; the scale bars are 40 µm in length. Reproduced with permission from [66], Copyright 2018, WILEY-VCH Verlag GmbH & Co. KGaA, Weinheim. (**b**) Illustration depicting reconfigurable multistate optical systems based on VO_2_ phase transitions modulated by temperature, e-doping, and H-doping. Schematic of stacked Al_2_O_3_/Al nanodisks on a VO_2_/Au mirror substrate with nanosized Pd dots, where the Pd dots were utilized to facilitate its hydrogenation and dehydrogenation. SEM images of a palette square. Scale bar: 200 nm. Optical micrographs of a color palette with stepwise tuning of diameter (D) and gap (g) at the four different states, monoclinic (m)-VO_2_, rutile (r)-VO_2_, H-VO_2_ (M), and H-VO_2_(I). Reproduced with permission from [67], Copyright 2020, American Chemical Society. (**c**) Self-organized gold network (SGN)–VO_2_ hybrid films for color filter application. Photographs of bare VO_2_ (80 nm) film (1) and SGN–VO_2_ hybrid films (2–4) for various Au thicknesses (VO_2_ thickness fixed at 80 nm). Photographs of SGN–VO_2_ hybrid films (5–8) for various VO_2_ thicknesses (Au thickness fixed at 50 nm). Reproduced with permission from [68], Copyright 2020, WILEY-VCH Verlag GmbH & Co. KGaA, Weinheim.

In addition to light transmission modulation of VO_2_, the device applications using dynamic color modulation based on the phase transition of VO_2_, in combination with nanostructured metals, were recently reported [66,67,68]. Shu et al. [66] demonstrated color generation for display and imaging applications through the integration of plasmonic nanostructures with periodic silver-nanodisk arrays on VO_2_ film (Figure 13a). As illustrated in Figure 13a, the reflection images of samples can readily be tuned by adjusting the geometric parameters (diameter and periodicity) of the nanodisks, and the color of the sample changes with the increase in nanodisk diameter at both 20 and 80 °C. In Figure 13a, the scanning electron microscopy (SEM) images for four different patterns of VO_2_ and periodic silver-nanodisk arrays showed distinctively different reflection color images of the patterns at 20 and 80 °C, indicating the realization of abundant color variation due to the MIT of VO_2_ and surface plasmon effect of metal nanostructures. Liu and co-workers demonstrated reconfigurable multistate optical systems enabled by phase transitions in VO_2_, which could be modulated by thermal tuning, hydrogen (H)-doping, and electron (e)-doping, as shown in Figure 13b [67]. Specifically, they presented a quadruple-state dynamic plasmonic display based on stacked structures of aluminum (Al)/Al_2_O_3_ nanodisks on a VO_2_/Au mirror substrate with Pd dots, in response to a combination of temperature and H-doping (Figure 13b, bottom panels). Meanwhile, In et al. [68] proposed composites of self-organized gold network (SGN) and VO_2_ as promising templates for photonic applications, combining advantages of both MIT hysteresis and strong light–matter interactions. They demonstrated thermoactive cyan–magenta–yellow color filters based on SGN–VO_2_ hybrid films, which were fabricated on 2 in. sapphire wafers with various VO_2_ thickness, as shown in Figure 13c.

## 4. Summary and Outlook

In this review, we first introduced several solution-based and gas-phase-based synthesis methods of nanostructured VO_2_ and modulation approaches of its properties, such as stoichiometry, strain (or stress), and doping. Among the potential applications of VO_2_ nanostructures and optical sensing devices, including photodetectors, optical switches, and color modulators, were discussed. Specifically, we reviewed and summarized photodetection mechanisms and VO_2_-based photodetectors for UV, visible, NIR, and IR lights, including optical transmission and dynamic color modulations. Many researchers have devoted to modulate the MIT properties and have demonstrated the design of various device applications.

In addition to the applications mentioned in this review, very recently, VO_2_ has been considered as one of the promising materials for energy-efficient neuromorphic computing applications, owing to the rise of artificial intelligence related to the fourth industrial revolution [23,70,71,72,73,74]. Moreover, VO_2_ has attracted much attention as a promising material for adaptive radiative cooling due to a thermochromic property, offering a potential way to reduce energy consumption in buildings [75,76,77]. However, there are still some challenges, such as scalable and reliable fabrication of nanostructured VO_2_ materials and precise control of their phase-transition properties (transition temperature, resistivity ratio, hysteresis, transition pathway, phase coexistence, etc.). Thus, it is still necessary to achieve a comprehensive understanding of the MIT in VO_2_, substantial progress for practical uses, and enhanced regulation of the physical and chemical processes associated with future research fields, including the applications discussed above. Finally, further advancements in VO_2_ will pave the way for new possibilities and opportunities, enabling the expansion of VO_2_-based devices into a wider range of innovative functional applications.

## Figures and Tables

**Table 1:** Synthesis methods of nanostructured VO_2_ [3,12,13,14,15].

Synthesis Method	Advantages	Limitations
Sol-gel	Simple, cheap, and precise control of variables Creation of amorphous materials High chemical reactivity of precursors	Post-deposition heat treatment Long preparation and curing times Poor adhesion
Hydrothermal synthesis	Easy regulation of size, shape, and composition Higher chemical purity	Expensive autoclaves Longer reaction times Poor adhesion
PLD ^1^	Growth of compatible and consistent films No restrictions on the types of PLD targets	Slow process and high cost preparation Not suitable for vast area deposition Material loss due to evaporation
Sputtering	Deposition of stable and uniform films Easy deposition of hybrid materials Good film adhesion	Expensive and sophisticated equipment Low purity
CVD ^2^	Growth of nanostructures with various shapes High-quality crystalline Small-scale crystals at low cost and in a short time Rapid and inexpensive method	Numerous growth parameters: growth time, temperature, precursors, ambient conditions, and substrate, etc. Influences of growth substrates: crystal size, shape, and orientation, etc.

^1^ PLD: pulsed laser deposition. ^2^ CVD: chemical vapor deposition.

## Data Availability

Not applicable.
